# Full Genome Characterization of Respiratory Syncytial Virus Causing a Fatal Infection in an Immunocompromised Patient in Tunisia

**DOI:** 10.3390/pathogens11070758

**Published:** 2022-07-02

**Authors:** Valentina Curini, Maurilia Marcacci, Salma Abid, Monia Ouederni, Awatef ElMoussi, Latifa Charaa, Wafa Achour, Ramzi Ouhichi, Latifa Maazaoui, Adriano Di Pasquale, Hakim ElGhord, Ahlem Gzara, Alessandro Ripani, Francesca Di Giallonardo, Cesare Cammà, Alessio Lorusso, Ilhem Boutiba-Ben Boubaker

**Affiliations:** 1 Istituto Zooprofilattico Sperimentale dell’Abruzzo e Molise (IZS-Teramo), 64100 Teramo, Italy; v.curini@izs.it (V.C.); a.dipasquale@izs.it (A.D.P.); a.ripani@oie.int (A.R.); c.camma@izs.it (C.C.); a.lorusso@izs.it (A.L.); 2 Department of Veterinary Medicine, University of Bari, 70010 Bari, Italy; 3 Laboratory «Antimicrobial Resistance»(LR99ES09), Faculty of Medicine of Tunis, University of Tunis El Manar, Tunis 1007, Tunisia; salma.abid@rns.tn (S.A.); awatef.elmoussi@rns.tn (A.E.); ilhem.boutiba@fmt.utm.tn (I.B.-B.B.); 4 National Influenza Center, Laboratory of Microbiology, Charles Nicolle Hospital, Tunis 1007, Tunisia; latifa.charaa@gmail.com; 5 Department of Peadiatrics: Immuno-Haematology and Stem Cell Transplantation, National Center of Bone Marrow Transplantation, Faculty of Medicine, Tunisia-University Tunis El Manar, Tunis 1006, Tunisia; monia.ouederni@fmt.utm.tn; 6 Laboratory Department, National Center of Bone Marrow Transplantation, Tunis 1006, Tunisia; wafa.achour@fmt.utm.tn; 7 Research Laboratory «Microbiology of Children and Immunocompromised»(LR18ES39), Faculty of Medicine, University of Tunis El Manar, Tunis 1006, Tunisia; 8 Tunisian Country WHO Office, Tunis 1003, Tunisia; ouhichir@who.int; 9 Ministry of Health, Primary Health Care Directorate, Tunis 1006, Tunisia; latifa.maazaoui@rns.tn (L.M.); hakim.elghord@rns.tn (H.E.); ahlem.gzara@rns.tn (A.G.); 10 OIE Sub-Regional Representation for North Africa, Tunis 2091, Tunisia; 11 The Kirby Institute, University of New South Wales (UNSW), Sydney, NSW 2052, Australia; fdigiallonardo@kirby.unsw.edu.au

**Keywords:** *Human orthopneumovirus*, next generation sequencing, whole genome, phylogenetic analysis, HRSV-A

## Abstract

*Human orthopneumovirus* (HRSV) is a virus belonging to the *Pneumovirus* genus that causes lower respiratory tract infections (LRTI) in infants worldwide. In Tunisia, thousands of infants hospitalized for LRTI are found to be positive for HRSV but no whole genome sequences of HRSV strains circulating in this country are available thus far. In this study, five nasal swab samples collected at different time points from a three-month-old female baby with severe immunodeficiency that was hospitalized for acute bronchiolitis were investigated by next generation sequencing. The Tunisian sequences from this study originated from samples collected in 2021, belong to the ON1 genotype of HRSV-A, and are clustered with European sequences from 2019 and not from 2020 or 2021. This is most likely related to local region-specific transmission of different HRSV-A variants due to the COVID-19 related travel restrictions. Overall, this is the first report describing the whole genome sequence of HRSV from Tunisia. However, more sequence data is needed to better understand the genetic diversity and transmission dynamic of HRSV.

## 1. Introduction

*Human orthopneumovirus* (HRSV), formerly known as respiratory syncytial virus (RSV), is the leading cause of lower respiratory tract infections (LRTI) in infants worldwide [1]. Morbidity and mortality are higher in premature infants, individuals with chronic respiratory diseases and severe combined immunodeficiency patients (SCID) [2]. Although a number of vaccines against HRSV are currently in late-stage clinical trials, none of them are available on the market. Ribavirin has been used for decades for treating RSV infections [3]. However, the high morbidity and mortality in high-risk populations, conflicting results in the clinical effectiveness and the high cost of ribavirin have underlined the need for a preventive strategy. Currently, the only pharmaceutical option is an mAb used for prophylactic treatment of high-risk children [4]. HRSV is an enveloped negative-sense, single-stranded RNA ((-) ssRNA) virus belonging to the *Paramyxoviridae* family, *Pneumovirus* genus. The genome of about 15 kb in length is composed of ten genes codifying two non-structural proteins (NS1 and NS2) and nine structural proteins (N, P, M, SH, G, F, M2-1, M2-2 and L) as for two overlapping open reading frames (ORFs) in the M2 gene [5]. The envelope of the virus contains two major glycoproteins: glycoprotein G (GPG) and glycoprotein F, which are essential for virus attachment and entry to the host cell. GPG, encoded by the G gene, is the most variable of the HRSV proteins and has been shown to accumulate mutations over time [1]. HRSV exists as a single serotype with two major antigenic subgroups, named HRSV-A and HRSV-B, which co-circulate in the field [6]. This classification is due to variations in one of the two hypervariable regions (HRV2) located at the C-terminal of the GPG. Based upon the HRV2 gene, at least 14 and 25 genotypes have been described globally for HRSV-A and HRSV-B, respectively [7]. In temperate regions, HRSV infections show a distinct seasonality with onset in late fall or early winter; usually the infection peak is between mid-December and early February [8]. The World Health Organization (WHO) piloted an HRSV surveillance strategy that leverages the existing capacities of the Global Influenza Surveillance and Response System (GISRS) to better understand HRSV seasonality, high-risk groups, validate case definitions, and develop laboratory and surveillance standards for HRSV globally [9]. However, the daily use of next generation sequencing (NGS)-based techniques in the HRSV milieu is limited. Indeed, the current COVID-19 pandemic highlighted once more the importance of NGS-based techniques as a fundamental tool helping scientists to study the SARS-CoV-2 mutations over time and genomic epidemiology [10,11]. Until recently, no whole genome sequence data for HRSV was available for infections in Tunisia. A recent study analyzed the molecular epidemiology of HRSV in Tunisia based on the analysis of 1417 nasopharyngeal aspirates collected from infants hospitalized for LRTI in five Tunisian hospitals between 2015 and 2018. Overall, 27.8% were diagnosed as positive for HRSV by a direct immunofluorescence assay. A further analysis of 61 randomly selected RSV strains revealed that HRSV-A (78.7%) predominated during the period of study as compared to HRSV-B (21.3%). The phylogenetic analysis, based on partial sequences of HVR2 of the GPG, the ON1 genotype (with a 72- nucleotide duplication in HVR2 of the G gene) was predominant (98.0% of HRSV-A strains), while one HRSV-A strain was clustered with the NA1 genotype (2.0% of HRSV-A strains). As for HRSV-B strains, all sequences contained a 60-nt insertion in HVR2 and clustered within the BA10 genotype [12]. In the study here, we report the first whole genome sequence of HRSV in Tunisia sampled in 2021 from a three-month-old baby with severe combined immunodeficiency admitted to the National Bone Marrow Transplant Center of Tunis for acute bronchiolitis.

## 2. Description of Clinical Case

A three-month-old female baby, born from consanguineous parents, was admitted in the Department of Pediatrics: Immuno-Haematology and Stem Cell Transplantation, at the National Bone Marrow Transplant Center of Tunisia with the diagnosis of acute bronchiolitis. Forty-eight hours before admission, she presented upper respiratory symptoms without fever. Physical examination revealed polypnea, bilateral crackles, intercostal retractions and hypoxemia and she required oxygen therapy. Furthermore, she had oral thrush, left axillar adenomegaly and diffuse cutaneous rush. Chest X rays showed bilateral alveolo-interstitial infiltrates and the absence of thymus. Chest tomography presented cellular bronchiolitis with bilateral micronodules and condensations. Blood analysis showed a low hemoglobin level (9 g/dL) (reference values: 9.5–14.1 g/dL), an eosinophils count of 4000/mm^3^ (reference values: 100–1000/mm^3^), absolute neutrophil count of 2300/mm^3^ (reference values: 1000–6000/mm^3^), an absolute lymphocyte count of 1560/mm^3^ (reference values: 4000–12,000/mm^3^), and a platelet count of 500,000/mm^3^ (reference values: 150,000–450,000/mm^3^). The C Reactive Protein (CRP) and procalcitonin were negative.

The FastTrack Diagnostics (FTD) respiratory pathogens 21 assay (Siemens, Esch-sur-Alzette, Luxembourg) was performed on nasopharyngeal swab samples for the molecular detection of respiratory pathogens including (adenovirus, bocavirus, low pathogenic human coronaviruses, enterovirus, human metapneumovirus A/B, parechovirus, influenza A, influenza A H1N1, influenza B, human parainfluenza virus 1–4, human respiratory syncytial virus (HRSV) A/B, rhinovirus, and *Mycoplasma pneumoniae*. The presence of *Pneumocystis jirovecii*, *Candida* spp., *Aspergillus* spp., and *Mycobaterium* spp. was investigated by culture of the bronchoalveolar lavage.

Serum immunoglobulin values were low (IgG 0.31 g/L; IgA 0.02 g/L; IgM 0.10 g/L). Immunophenotyping on flow cytometry showed the following results: CD3^+^ T cells: 205 cells/mm^3^; CD4^+^ T cells: 102/mm^3^; CD8^+^ T cells: 28/mm^3^; CD19^+^ B cells: 16/mm^3^ and CD56^+^ CD3^−^ NK cells: 1420/mm^3^, compatible with a SCID T−B-NK+. Hypereosinophilia and cutaneous biopsy analysis were compatible with an Omenn syndrome, thus, intravenous immunoglobulin substitution was commenced. Based on the immunodeficiency status, severity of respiratory signs and adenomegaly of the left axillary suggesting a Bacille Calmette-Guérin (BCG) disease (a rare life-threatening complication of BCG vaccine administration), a wide-spectrum antibiotic treatment was administered. Unfortunately, the worsening of respiratory distress required nasal high flow oxygen (NHF) therapy. Chest tomography revealed an increment of bilateral infiltrates and the FTD respiratory pathogens assay turned out positive for HRSV. The patient received regular immunoglobulin replacement with oral ribavirin and without Palivizumab. A haploidentical hematopoietic stem cell transplantation (haplo-HSCT) was performed when nasal high flow oxygen was stopped, and severe respiratory distress was resolved. However, the child remained symptomatic with mild polypneathus requiring oxygen therapy. A reduced intensity conditioning (RIC) regimen included Fludarabine and low doses of Busilvex. GVHD prophylaxis involved in vivo T cell depletion by post-transplant cyclophosphamide, cyclosporin and mycophenolate mofetil. She continued to receive wide spectrum antibiotics treatment. Although engraftment was successful, the respiratory signs worsened in concomitance with the persistence of HRSV on nasal swabs, even after HSCT. The patient died with acute respiratory distress four weeks after HSCT and three months after the onset of initial symptoms.

## 3. Materials and Methods

### 3.1. Ethics

All procedures performed in the study were in accordance with the ethical standards of the Charles Nicolle Hospital of Tunis (Tunisia) and with the 1964 Helsinki declaration and its later amendments. Informed consent was obtained from the parents of the individual participant included in the study.

### 3.2. Samples

Five nasal swab samples collected at different time points (11 May 2021, 19 May 2021, 1 June 2021, 16 June 2021, and 16 July 2021) were sent to the Virology unit of the Microbiology lab of Charles Nicolle Hospital. All were tested for the presence of HRSV RNA by an FTD Respiratory Pathogens 21 assay. The cycle threshold (C*_T_*) values were 15.37, 18.38, 24.46, 16.48 and 18, respectively.

### 3.3. Next Generation Sequencing

NGS analysis was conducted at IZS-Teramo for all RNA samples purified from the collected nasal swabs positive for HRSV RNA. Total RNA was subjected to Turbo DNase treatment (Thermo Fisher Scientific, Waltham, MA, USA) at 37 °C for 20 min and then purified by RNA Clean and Concentrator-5 Kit (Zymo Research, Irvine, CA, USA). The purified RNA was used for the assessment of sequence independent single primer amplification protocol (SISPA) using SuperScript^®^ IV Reverse Transcriptase (Thermo Fisher Scientific, Waltham, MA, USA) with the random-tagged primer FR26RV-N 5′- GCCGGAGCTCTGCAGATATCNNNNNN-3′ [13]. The PCR products were purified by ExpinTM PCR SV (GeneAll Biotechnology CO., Seoul, Korea) and then quantified using the Qubit^®^ DNA HS Assay Kit (Thermo Fisher Scientific, Waltham, MA, USA). Libraries were prepared using Illumina^®^ DNA Prep, (M) Tagmentation (96 Samples) (Illumina Inc., San Diego, CA, USA) according to the manufacturer’s protocol. Deep sequencing was performed on the NextSeq500 (Illumina Inc., San Diego, CA, USA) using the NextSeq 500/550 Mid Output Reagent Cartridge v2 (300 cycle) (Illumina Inc., San Diego, CA, USA) and standard 150 bp paired-end reads. After a quality check and trimming of raw reads data using FastQC v0.11.5 and Trimmomatic v0.36, respectively, host depletion was performed by Bowtie2 [14]. De novo assembly was carried out using SPAdes v3.11.1 [15] and the contigs obtained were compared by BLASTn analysis in order to identify the closest publicly available sequence in the GenBank database. Several contigs showed high percentage of identity (98.39%) with HRSV-A strain OX02-9571-V01 (GenBank accession number MZ515567) that was then used as reference to perform mapping by BWA software package (v.0.7.17) [16]. The HRSV consensus sequences was obtained by iVar (v1.3.1) (intrahost variant analysis of replicates; github.com/andersen-lab/ivar).

### 3.4. Sequence Alignments and Phylogenetic Analysis

Global sequence data for HRSV-A was downloaded from the GISAID EpiRSVTM database (https://www.gisaid.org/, accessed on 7 February 2022), and the NCBI (https://www.ncbi.nlm.nih.gov/nucleotide/, accessed on 7 February 2022), and Virus Pathogen Database and Analysis Resource (ViPR) (https://www.viprbrc.org/). Full-genome sequences were aligned in MAFFT implementing the FFT-NS-2 algorithm [17,18]. The alignment was manually inspected in Geneious to ensure accuracy and that identical sequences were removed. The final global data set consisted of 2493 HRSV-A nucleotide sequences. These were combined with sequences from the patient in this study. Of note, some of the patient sequences had low coverage and thus many ambiguities (~14%) and were omitted from the full-genome phylogeny. An HRSV-B sequence was used as an outgroup (GenBank accession: MT107528). The HRV2 of the G gene (336 bp) was extracted from the global alignment and combined with the HRSV-A sequences from Jerbi et al. [7] Identical global sequences were again removed, and the final data set consisted of 1070 sequences, including 36 sequences from Jerbi et al., and five sequences from the study here. Phylogenetic trees for the full genome were estimated using FastTree [19,20]. To simplify visualization a random subset of 300 partial G genes from the global data was extracted and combined with Tunisian sequences. This reduced cleaned data set contained 342 sequences. These G gene phylogenetic trees were estimated in IQTree [21] implementing ModelFinder [22] and 1000 ultrafast bootstrap replicates [23]. Trees were visualized in FigTree v1.4.4 (https://github.com/rambaut/figtree/releases, accessed on 7 February 2022).

## 4. Results and Discussion

**Figure 1:**
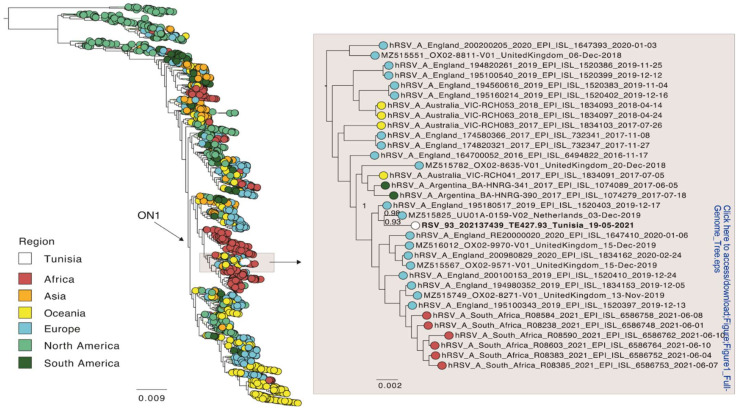
Global phylogeny of full-genome sequences of HRSV-A. Global sequences were combined with a sequence from this study (white) and a phylogenetic tree was estimated using FastTree. The position of the Tunisian sequence is shown enlarged. The sequences are colored according to the geographic region they were sampled in. Branch length indicates nucleotide substitutions per site, and node support is indicated.

Of the five time points sampled, read coverage was enough to reconstruct a full-length consensus sequence of HRSV-A only for time points 2 (19 May 2021) and 4 (16 June 2021). These two sequences were identical. In addition, the five sequences obtained from the patient were identical to each other for those genomic regions that contained enough read coverage for a consensus comparison (~9800 nt). Thus, there was no evidence for within host evolution. Only the sequence of time point 2 was included in a global phylogenetic analysis and was shared with the NCBI database (acc. no. ON469827). This full genome phylogeny placed the patient’s sequence within the ON1 genotype for HRSV-A (Figure 1), and which is supported by the presence of the characteristic duplication of 72 nt in the G gene. The Tunisian strain from this study was most closely related to an isolate from the Netherlands from 2019 (NCBI accession MZ515825) with an overall nucleotide identity of 99%. These sequences differed by only nine non-synonymous substitutions, and none were present in the G or F gene (2 in NS2, 2 in M2, 5 in L).

**Figure 2:**
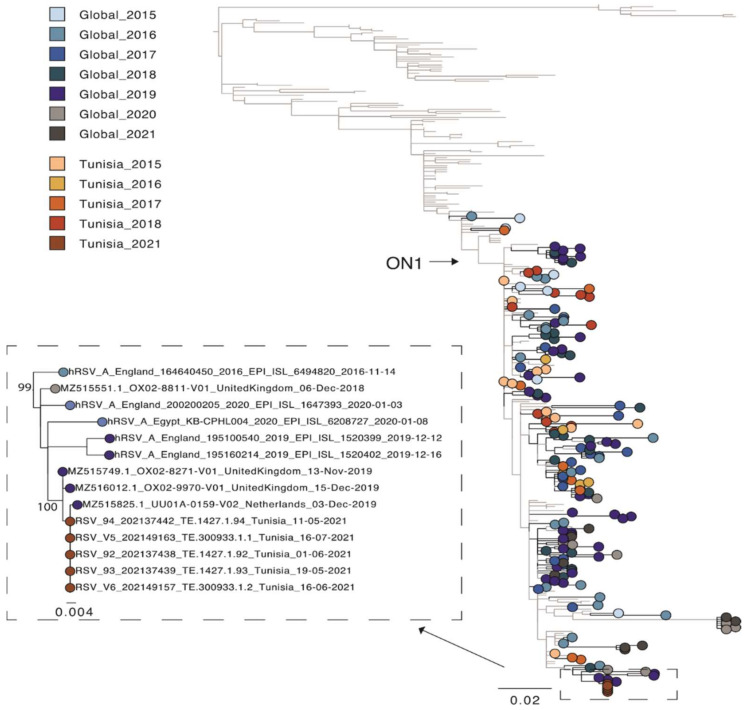
Phylogenetic tree of the hypervariable region in the G gene of HRSV-A sequences. A random selection of global sequences was used for the phylogeny including Tunisian sequences (*n* = 342). The tree is coloured according to year of sampling with colours getting darker from 2015 to 2021. Global sequence data is highlighted in blue and grey and Tunisian sequence data in red. Sequences from the study here are shown in black. An extraction of the node containing these latter sequences is also shown. Branch length indicates nucleotide substitutions per site and node support is shown.

A second phylogenetic tree containing other Tunisian sequences [7] was estimated for the partial G gene region only (as only data for this region was available). For this phylogeny, sequences for all five isolates from the patient were used (Figure 2). The patient’s strain clearly clustered together, as they were identical. The publicly available Tunisian sequences were sampled from outbreaks between 2015–2018 and were scattered across the phylogeny. This is characteristic of the new strains outcompeting older strains as multi-year persistence of HRSV is less common [6]. However, the Tunisian sequences from our study, which were sampled in 2021, clustered with European sequences from 2019 and not 2020 or 2021. This is most likely related to local region-specific transmission of different HRSV-A variants due to the COVID-19 related travel restrictions. However, more sequence data is needed to better understand the genetic diversity and transmission dynamic of HRSV present in Tunisia. In this regard, an HRSV-full genome-based surveillance analysis is currently ongoing in Tunisia. In conclusion, this study reports the first full genome characterization of HRSV from Tunisia. One of the primary advantages NGS provides over other methods of analysis is that it is an effective and high-throughput solution for screening multiple samples and detecting viruses without prior knowledge of an infectious agent. Among its core capabilities, NGS workflows can be used to detect HRSV virus, perform surveillance and epidemiological studies, track mutational changes in the virus and analyze potential susceptibility and human response to HRSV infection.

## Data Availability

Sequence data of the occurring HRSV-A strain is available from NCBI with the acc. no. ON469827 (https://www.ncbi.nlm.nih.gov/).
